# The use of single armed observational data to closing the gap in otherwise disconnected evidence networks: a network meta-analysis in multiple myeloma

**DOI:** 10.1186/s12874-018-0509-7

**Published:** 2018-06-28

**Authors:** Susanne Schmitz, Áine Maguire, James Morris, Kai Ruggeri, Elisa Haller, Isla Kuhn, Joy Leahy, Natalia Homer, Ayesha Khan, Jack Bowden, Vanessa Buchanan, Michael O’Dwyer, Gordon Cook, Cathal Walsh

**Affiliations:** 1 Department of Population Health, Luxembourg Institute of Health, Strassen, Luxembourg; 2 Department of Psychiatry, Trinity College Dublin, Dublin, Ireland; 3 Department of Psychology, University of Cambridge, Cambridge, UK; 4 Cogentia Healthcare Consulting, Cambridge, UK; 5 Department of Psychology, University of Zurich, Zürich, Switzerland; 6 University Library: Medical Library, University of Cambridge, Cambridge, UK; 7 Department of Computer Science and Statistics, Trinity College Dublin, Dublin, Ireland; 8 University of Exeter, Exeter, UK; 9 School of Social and Community Medicine, University of Bristol, Bristol, UK; 10 Department of Medicine, NUI Galway, Galway, Ireland; 11 Professor of Haematology & Myeloma Studies, Clinical Director NIHR MIC-DEL, St James’s University Hospital, Leeds, England; 12 Health Research Institute, University of Limerick, Limerick, Ireland; 13 Department of Health Policy & Management, Mailman School of Public Health, Columbia University, New York, USA

**Keywords:** Network meta-analysis, Single armed studies, Evidence synthesis, Relapsed or refractory myeloma

## Abstract

**Background:**

Network meta-analysis (NMA) allows for the estimation of comparative effectiveness of treatments that have not been studied in head-to-head trials; however, relative treatment effects for all interventions can only be derived where available evidence forms a connected network. Head-to-head evidence is limited in many disease areas, regularly resulting in disconnected evidence structures where a large number of treatments are available. This is also the case in the evidence of treatments for relapsed or refractory multiple myeloma.

**Methods:**

Randomised controlled trials (RCTs) identified in a systematic literature review form two disconnected evidence networks. Standard Bayesian NMA models are fitted to obtain estimates of relative effects within each network. Observational evidence was identified to fill the evidence gap. Single armed trials are matched to act as each other’s control group based on a distance metric derived from covariate information. Uncertainty resulting from including this evidence is incorporated by analysing the space of possible matches.

**Results:**

Twenty five randomised controlled trials form two disconnected evidence networks; 12 single armed observational studies are considered for bridging between the networks. Five matches are selected to bridge between the networks. While significant variation in the ranking is observed, daratumumab in combination with dexamethasone and either lenalidomide or bortezomib, as well as triple therapy of carfilzomib, ixazomib and elozumatab, in combination with lenalidomide and dexamethasone, show the highest effects on progression free survival, on average.

**Conclusions:**

The analysis shows how observational data can be used to fill gaps in the existing networks of RCT evidence; allowing for the indirect comparison of a large number of treatments, which could not be compared otherwise. Additional uncertainty is accounted for by scenario analyses reducing the risk of over confidence in interpretation of results.

**Electronic supplementary material:**

The online version of this article (10.1186/s12874-018-0509-7) contains supplementary material, which is available to authorized users.

## Background

Network meta-analysis (NMA) has become increasingly popular among both clinicians and policy makers as a tool to assess the evidence for new technologies relative to all available comparator treatments [1]. The technique allows researchers to estimate the comparative effectiveness of treatments that have not been studied in head to head trials. However, relative treatment effects for all interventions of interest can only be derived where it is possible to establish a viable, connected network (see Lu and Ades for an introduction [2]). Unfortunately, it is often challenging to find high quality evidence (e.g. RCT) for all potentially relevant treatments of interest, and as a result evidence networks may be partial or incomplete.

One option is to conclude that evidence is insufficient to make a judgement on relative treatment effects. Often, however, a decision on reimbursement or treatment choice is required and cannot be postponed. One could rely on clinical judgement to inform the comparative effects, as has been done in the past, however, additional uncertainty is not being accounted for [3]. Recently, novel methods have been proposed as a means of incorporating evidence from observational studies or patient level data and thereby potentially overcoming some of the limitations described above. Hierarchical models have been proposed to systematically incorporate comparative observational evidence based on summary as well as individual patient level data [4–6]. Random main effects models allow for the incorporation of before-and-after studies, where access to patient level data is not a necessity [7]. An alternative is to simultaneously synthesise multiple outcome measures and derive relative effects through a chain of evidence [8]. Complex methods such as propensity scoring or matching adjusted indirect comparison make use of individual patient level data to create a comparison adjusting for measured covariates [9–13]. The choice of method depends on the data available. RCTs continue to be the gold standard of evidence. Analyses based on individual patient level data allow for the adjustment of observed covariates; however, individual patient level data is quite often unavailable. Analyses based on summary data are prone to bias and need to be interpreted with great care.

Multiple myeloma (MM) is the second most common form of blood cancer with an age-adjusted incidence of six per 100,000 per year in the USA and Europe [14, 15]. Initial treatment options for MM typically involve corticosteroids in combination with other drugs including alkylating chemotherapeutic agents and novel biological drugs, with or without hematopoietic stem cell rescue [15, 16]. Several, novel biological drugs have demonstrated promising activity in treating MM including immunomodulatory drugs (e.g. thalidomide, lenalidomide and pomalidomide) and proteasome inhibitors (e.g. bortezomib and carfilzomib). Yet there continues to be a substantial unmet clinical need, and at present there is no cure for MM with relapse remaining inevitable [17, 18]. Given the poor prognosis for relapsed and refractory MM (RRMM), there is an immediate demand to establish effective, evidence-based treatment approaches in this area of unmet clinical need. Currently, comprehensive comparative data between treatments and disease stages is lacking [19]. An assessment of clinical effectiveness across pharmacological treatments for RRMM is essential in order to establish how treatments for RRMM compare on outcomes.

There are a number of non-systematic reviews available which discuss the utility of available or emerging treatments for RRMM [17, 20, 21]. Previous systematic reviews have tended to focus on single drugs [22–25]; few considered survival outcomes and the clinical effectiveness of more than one drug intervention for RRMM. Lopuch et al. [26] used data from four RCTs to evaluate the safety and efficacy of targeted pharmacological interventions for RRMM, used as monotherapy or in combination with other drugs. Dranitsaris and Kuara offer an indirect comparison of lenalidomide and bortezomib specifically using data from three RCTs [27]. However, these papers are limited in scope and do not encompass the broad variety of active treatments available for RRMM.

More recently, three more comprehensive analyses were published. The Institute for Clinical and Economic Review report on treatments for RRMM presented a NMA comparing the relative effectiveness of seven interventions using data from randomised and single-arm studies [28]. Disconnected evidence was linked through a comparison of two key treatment regimens (bortezomib plus dexamethasone and bortezomib monotherapy) obtained from a retrospective matched pairs analysis [29]. Van Beurden-Tan and colleagues obtained relative effects for 18 treatment options under the assumption of equal efficacy of bortezomib plus dexamethasone and bortezomib monotherapy as well as thalidomide plus dexamethasone and thalidomide monotherapy [3]. Armoiry et al. have recently highlighted the disagreement between published matched pair analyses and the assumption of equal efficacy applied here [30]. Botta et al. obtained relative effects across the network by grouping regimens into nine groups [31]. An analysis of independent treatments is also provided, however, still assuming equal efficacy of bortezomib monotherapy and bortezomib plus dexamethasone as well as thalidomide, thalidomide plus dexamethasone and lenalidomide plus dexamethasone. None of these analyses incorporated the additional uncertainty introduced by making assumptions of equal efficacy or using estimates obtained through retrospective analysis.

The objective of this analysis is to fill the gap in RCT evidence by utilising additional information from observational evidence to obtain relative effect estimates of all treatments for RRMM, while capturing additional uncertainty to avoid over confidence in interpretation of results.

## Methods

### Literature search and data extraction

A systematic search of the published literature and relevant conference proceedings was conducted to identify eligible studies and is reported following PRISMA guidelines. The review protocol is published in Prospero (www.crd.york.ac.uk/PROSPERO/display_record.asp?ID=CRD42014013405). In August 2014, the first search was carried out in MEDLINE, EMBASE and the Cochrane Library’s Central Register of Controlled Trials; the search was updated in January 2016 and February 2017 (RCTs only). Papers were first checked by title, and then underwent abstract review. Papers were required to be in English and were included if they presented (a) original studies, (b) clinical effectiveness of any (pharmacological) intervention for the treatment of RRMM, and (c) reported progression-free survival (PFS), overall survival (OS) or time to progression (TTP) as primary or secondary outcome. The analysis presented here focuses on median PFS, study details are therefore restricted to studies reporting this outcome. Phase I dose-escalation studies, studies focusing on patient samples with different or mixed treatment conditions, and studies presenting subgroup analysis of a dataset adopted from a main clinical trial were excluded. For RCTs, conference abstracts and presentations were excluded if a corresponding published paper was available or could be identified snowballing. Conference abstracts for observational studies were excluded as they were limited in intervention and outcome information and lacked evidence of scientific validation. The full electronic search strategy can be accessed in online under Additional file 1. The quality of trials included in the NMA was assessed using the Cochrane risk of bias tool from the Cochrane handbook for RCTs [32] (ÁM, JL) and an adapted Newcastle Ottawa Scale (NOS) for observational studies [33] (JL, NH). Three authors (ÁM, EH, VB) extracted data on population characteristics, intervention description, and outcome measures. Estimates of the relative effectiveness of treatments on the hazard ratio scale, along with a measure of precision (standard error) were extracted, as well as median time to event data for each trial arm.

### Statistical analysis

#### RCT only analysis

In a first step, standard Bayesian NMA models were fitted to analyse RCT evidence only. NMA models provide a powerful method to synthesize data from multiple trials and generate estimates of relative efficacy between treatments within connected networks of evidence, by combining direct and indirect evidence [34]. Indirect estimates rely on the assumption of transitivity and the use of relative effects ensures randomisation is preserved.

Based on median PFS data and patient numbers, the model estimated the relative efficacy for each pairwise comparison, measured as hazard ratios (HRs) assuming an exponential survival model.

For each arm k in study i, a binomial likelihood function is used to model the number of patients alive at median time to event *r*_*i*, *k*_ , out of a total number of patients included in the arm *n*_*i*, *k*_.

$$ {r}_{i,k}\sim bin\left({p}_{i,k},{n}_{i,k}\right) $$

Based on the estimated survival probability *p*_*i*, *k*_ the model estimates the log hazard *loga*_*i*, *k*_ using the median time to event *w*_*i*, *k*_ and assuming an exponential survival function.

$$ {p}_{i,k}=1-\exp \left(-{w}_{i,k}\cdot \exp \left({loga}_{i,k}\right)\right) $$

Using standard NMA modelling, the model then estimates log hazard ratios compared to baseline treatments in each trial (*δ*_*i*, *k*_).

$$ {loga}_{i,1}={\mu}_i+0 $$

$$ {loga}_{i,k}={\mu}_i+{\delta}_{i,k}\kern3em ,k\ne 1 $$

PFS was chosen as the preferred outcome as it was most widely reported among the included trials. TTP was used where PFS was not reported. Since this paper focusses primarily on the methodology, no additional survival outcomes were considered. Median PFS was used in order to accommodate the incorporation of observational studies, the majority of which do not report HRs for survival outcomes. The model was fitted in WinBUGs using the R2Winbugs package in R [35, 36]. A Bayesian approach was taken using non-informative prior distributions. Fixed effects were assumed due to the limited amount of trials comparing the same two interventions. The WinBUGs code is available as Additional file 2.

Bayesian analyses capture uncertainty in the form of posterior distributions. We summarised outcomes as means and 95% credible intervals for hazard ratios. Further, we established a ranking of alternative treatments based on the surface under the cumulative ranking curve (SUCRA) score [37]. The SUCRA score is defined as the normalised area under the curve of the cumulative ranking plot, which shows, for every treatment, the probability of being the best, among the two best, among the top three treatments, etc. for the range of available treatments. The SUCRA score ranges from 0 to 1, where 1 reflects the best treatment with no uncertainty and 0 reflects the worst treatment with no uncertainty.

#### Extending NMA with observational studies

RCT data in this analysis formed two disconnected treatment networks, making comparisons of treatments between networks impossible using standard techniques. The aim of incorporating observational data here is to strengthen the existing RCT data and assist in drawing comparisons across all treatment interventions.

The analysis limited the inclusion of observational studies to those investigating at least one intervention, which was part of the RCT network. This restriction resulted in the exclusion of all potentially relevant comparative observational studies, leaving only single armed studies for inclusion. In the absence of access to patient level data, single armed observational trials were matched to act as each other’s control group based on covariate information. The inclusion of single armed studies was hence restricted to those reporting a complete covariate profile. Only studies investigating different interventions were considered as potential matched pairs.

A clinical expert in MM provided guidance for identifying and ranking covariates relevant for predicting treatment outcomes (MOD). Covariates selected in descending order of importance were: Frailty (defined by a composite of age, Charlson’s comorbidity score (CCS) and activity daily score (ADS)); genetic risk profile, treatment history, baseline stage and gender. Age was used as surrogate for frailty, since CCS and ADS were not generally reported in the trials. Genetic risk profile information was also very rarely reported and therefore not included. Finally, we used treatment history (weight = 4, measured as the medium number of prior treatments; normalised assuming a range of 0–4 prior lines), age (weight = 3, measured as median age, normalised assuming a range of 20–80 as median age), baseline stage (weight = 2, measured as mean baseline stage, normalised assuming a range of 0–3) and gender (weight = 1, measured as the proportion of females in each study). The distance *∆*_*tot*_ between any two studies j and k was determined as the weighted average of differences in covariates:

$$ {\Delta}_{tot}\left[j,k\right]=\frac{\sum \limits_{i=1}^4{w}_i\cdot {\Delta}_i\left[j,k\right]}{\sum \limits_{i=1}^4{w}_i} $$

Where *w*_*i*_ refer to the weights given to individual covariates and *∆*_*i*_[*j*, *k*] represents the normalised difference between studies j and k in covariate i. A numerical example illustrating the process is provided as Additional file 3. The distance takes a value between 0 and 1, where small values indicate more similar trials. There is no guidance available as to what is an adequate threshold for similarity; a distance of 0.1 was selected as the maximum distance allowable for matching study pairs. The impact of varying the threshold in this application is reported elsewhere [38]. As a further investigation into the appropriateness of the threshold we have compared the distance between observational studies to distances between and within RCTs.

A base case model was fitted including all matches connecting the separate networks using the same modelling approach as described above. Further, each match was investigated separately incorporating the RCT evidence above as well as each match in turn. Investigating the range of possible matches this way allows for the evaluation of variation associated with matched trial approaches.

We validated our method by comparing our analysis with estimates from previous inter network comparisons [3, 29, 31, 39].

Each NMA model discarded 50,000 burn-in iterations and was run with 100,000 iterations and three chains. Visual inspection of chains and autocorrelation plots confirmed convergence and the effective sample size was checked.

## Results

### Study details

**Fig. 1:**
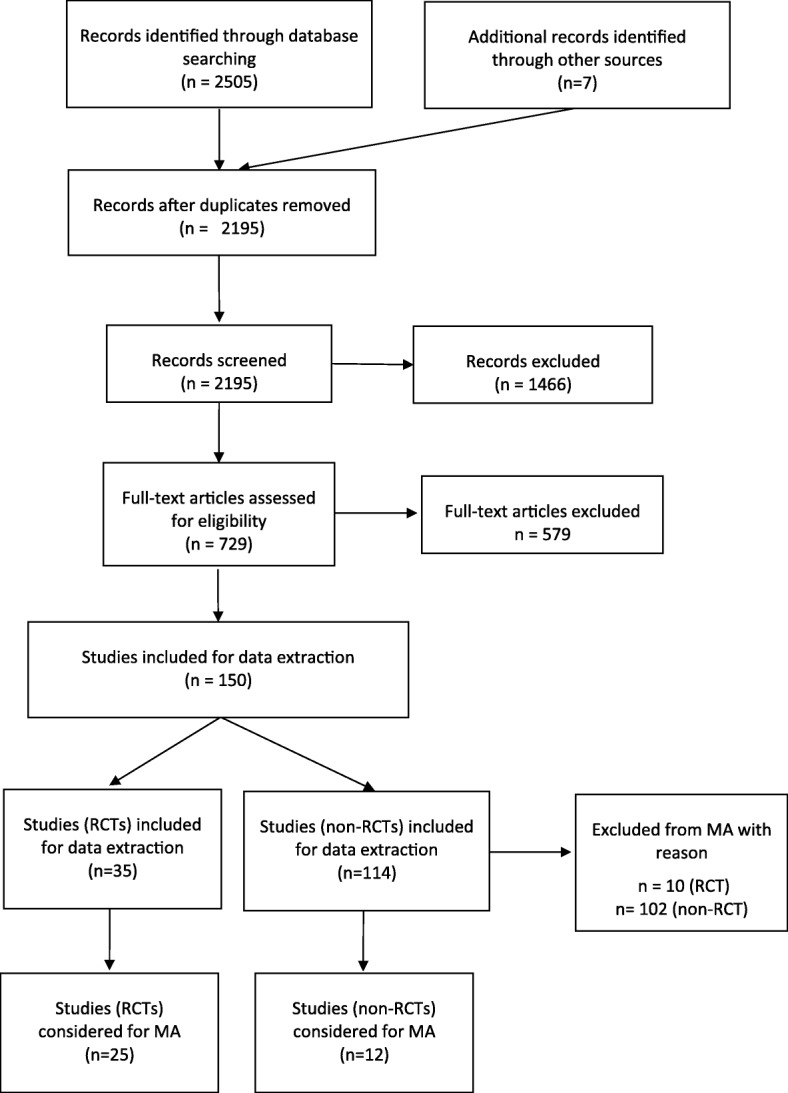
PRISMA flowchart

In total, 2505 papers were identified. After duplicates were removed, 2195 remaining titles and abstracts were screened for relevance. Of those, 1466 papers were excluded leading to 729 studies eligible for full-text reading. In total, 36 RCTs and 114 observational studies fulfilled the inclusion criteria and were used for data extraction. The PRISMA diagram is shown in Fig. 1. Excluding studies which did not report median PFS or TTP, studies investigating different doses or delivery methods of the same intervention, as well as observational studies investigating interventions not part of the RCT network or with incomplete covariate profile for single armed studies, resulted in 25 RCTs and 12 observational studies relevant for the analysis presented here. Reasons for exclusion of the remaining studies are presented in Additional file 4.

**Fig. 2:**
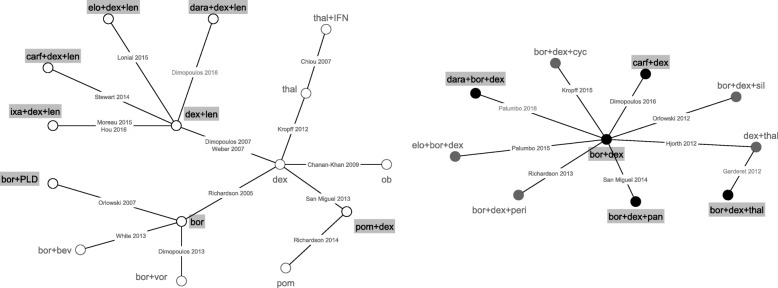
RCT evidence network: Each node represents a treatment regimen and connections between nodes indicate comparative RCT evidence. Interventions licensed in Europe are highlighted in grey. bev = bevacizumab; bor = bortezomib; carf = carfilzomib; cyc = cyclophosphamide; dara = daratumumab; dex = dexamethasone; elo = elozumatab; IFN = interferon alpha; ixa = ixazomib; len = lenalidomide; ob = oblimersen; pan = panobinostat; peri = perifosine; PLD = pegylated liposomal doxorubicin; pom = pomalidomide; sil = silituximab; thal = thalidomide; vor = vorinostat

Demographic information on the trials included in the analysis is shown in Table 1; the evidence network based on RCT evidence is shown in Fig. 2.

**Table 1:** Study characteristics of trials included in the analysis

Authors	Treatment	N	Median PFS (months)	Median Age (years)	Median number of previous lines	Mean baseline disease stage^b^	Female (%)
RCT trials							
Chanan-Khan et al. [48]	1. ob7mg + dex40mg	110	3.09	62	3	2.6	46
	2. dex40mg	114	3.55				
Chiou et al. [49]	1. thal100mg + IFN	16	1.5	63^c^	1	NR	14
	2. thal100mg	12	7.9				
Dimopoulos et al. [50]	1.bor1.3 mg/m2 + vor400mg	317	7.63	62	2	1.9	41
	2. bor1.3 mg/m2	320	6.83				
Dimopoulos et al. [51]	1. len25mg + dex40mg	176	11.3	63	NR	2.6	41
	2. dex40mg	175	4.7				
Dimopoulos et al. [52]	1. carf20mg + dex20mg	464	18.7	65	2	1.8	51
	2. bor1.3 mg/kg + dex20mg	465	9.4				
Hjorth et al. [53]	1. thal50mg + dex40mg	67	9	71	NR	NR	47
	2. bor1.3 mg/m2 + dex20mg	64	7.2				
Kropff et al. [54]	1. dex40mg	126	6	64	1–3 prior lines	1.9	57
	2. thal100/200/400 mg^a^	373	7.4				
Kropff et al. [55]	1. bor1.3 mg/kg + dex20mg	43	12.6	71	1	2.1	43^d^
	2. bor1.3 mg/kg + dex20mg + cyc50mg	47	9.9				
Lonial et al. [56]	1. elo10mg/kg + len25mg + dex40mg	321	19.4	66	2	1.8	40.4^d^
	2.len25mg + dex40mg	325	14.9				
Moreau et al. [57]	1. ixa4mg + len25mg + dex40mg	360	20.6	66	1	NR	43^d^
	2. len25mg + dex40mg	363	14.7				
Orlowski et al. [58]	1. sil6mg + bor1.3 mg/m2 + dex40mg	142	8.1	62	1–3 prior lines	NR	NR
	2. bor1.3 mg/m2 + dex40mg	144	7.6				
Orlowski et al. [59]	1. bor1.3 mg/m2	322	6.5	61	NR	NR	44
	2. PLD20mg + bor1.3 mg/m2	324	9				
Palumbo et al. [60]	1.elo10mg + bor1.3 mg/m2 + dex20mg	77	9.9	66	1^d^	NR	48^d^
	2. bor1.3 mg/m2 + dex20mg	75	6.8				
Richardson et al. [61]	1. pom4mg	108	2.7	63	5	2.6	46
	2. pom4mg + dex40mg	113	4.2				
Nagler et al. [62]	1.peri50mg + bor1.3 mg/m2 + dex20mg	69	5.23	NR	1^d^	NR	68
	2. bor1.3 mg/m2 + dex20mg	66	8.29				
Richardson et al. [63]	1. bor1.3 mg/m2	333	6.22	61	2	NR	42
	2. dex40mg	336	3.49				
San Miguel et al. [64]	1.pan20mg + bor1.3 mg/m2 + dex20mg	387	12	63	1	1.8	47
	2. bor1.3 mg/m2 + dex20mg	381	8.1				
San Miguel et al. [14]	1. pom4mg + dex40mg	302	4	64	5	2	41
	2. dex40mg	153	1.9				
Stewart et al. [65] [65]	1.carf20-17 mg + len25mg + dex40mg	396	26.3	64	2	NR	44
	2. len25mg + dex40mg	396	17.6				
Weber et al. [66]	1. len25mg + dex40mg	177	11.1	63	1–3 prior lines	2.5	40
	2. dex40mg	176	4.7				
White et al. [67]	1. bor1.3 mg/m2	53	5.1	65	1–3 prior lines	2.1	42
	2. bor1.3 mg/m2 + bev400mg	49	6.2				
Hou et al. [68]	1. ixa + len + dex	57	6.7	NR	2	1.7	31
	2. len + dex	58	4.0				
Dimopoulos et al. [69]	1. dara16mg/m2+ len25mg + dex40mg	286	54.1	65	1	1.7	NR
	2. len25mg + dex40mg	283	18.4				
Garderet et al. [70]	1. bor1.3 mg/m2 + thal200mg + dex40mg	135	18.3	61	1	1.6	37
	2. thal200mg + dex40mg	134	13.6				
Palumbo et al. [71]	1. dara 16 mg/m2 + bor1.3 mg/m2 + dex20 mg	251	18.5	64	2	1.8	43
	2. bor1.3 mg/m2 + dex20 mg	247	7.2				
Observational trials							
Avet-Loiseau et al. [40]	dex40mg + len25mg	207	9.6	65	3	1.7	44
Chang et al. [41]	bor1.3 mg/m2	65	9.5	54	> = 2	1.3	31
Fukushima et al. [72]	bor1.3 mg/m2 + dex20mg	22	16.8	69	2	2.1	41
Hou et al. [73]	dex20/40 mg + len25mg	199	8.3	60	4	2.8	47
Kneppers et al. [42]	dex40mg + len25mg	117	10	61	3.5	2.7	37
Lacy et al. [74]	dex40mg + pom2mg	34	4.8	62	4	1.7	32
Moore et al. [43]	bor1.3 mg/m2	52	13	72	1	2.6	40
Oehrlein et al. [75]	Dex < 160-480 mg	26	11.6	71	2	2.8	54
	Len10-25 mg						
Pantani et al. [76]	bor1.3 mg/m2 + dex20mg	85	8.7	58	2	2.4	40
Richardson et al. [77]	bor1.3 mg/m2 + dex40mg + pan20mg	55	5.4	61	4	1.9	47
Terpos et al. [78]	dex40mg + thal200mg	35	8	63	2	2.2	34
Walter-Croneck et al. [44]	bor1.3 mg/m2	708	14	60	1	2.1	55

*bev* bevacizumab, *bor* bortezomib, *carf* carfilzomib, *cyc* cyclophosphamide, *dara* daratumumab, *dex* dexamethasone, *elo* elozumatab, *IFN* interferon alpha, *ixa* ixazomib, *len* lenalidomide, *ob* oblimersen, *pan* panobinostat, *peri* perifosine, *PLD* pegylated liposomal doxorubicin, *pom* pomalidomide, *sil* silituximab, *thal* thalidomide, *vor* vorinostat

^a^Pooled results from 3 arms investigating different doses

^b^estimated excluding patients with unknown baseline stage

^c^probably not median but does not state exact method (presumably mean and SD)

^d^median

### Study quality

The RCTs were of mixed quality. Many studies were un-blinded, which created a high risk of bias. Additionally, the majority of studies failed to give sufficient information regarding randomisation and allocation concealment to determine the risk of selection bias. In most cases attrition bias was treated appropriately, and only one study presented a high risk, while another presented unclear risk in this regard. All but one of the studies were subject to high risk of bias due to other factors not accounted for in the Cochrane tool, such as sponsor involvement in study design, data collection and analysis and writing, small sample size, and by being a conference abstract rather than a full text peer reviewed paper. The observational studies showed a low risk of bias, with no study scoring below 4 out of a possible 6 stars. Details on the bias assessment are provided as Additional file 5.

### Analysis of RCTs only

Twenty-five RCTs investigating 25 separate treatment regimens were analysed. Of these regimens, 13 treatment combinations are currently licensed in Europe. Since comparisons of these interventions may be of primary interest, we have highlighted these in our results.

The combined RCT evidence forms two separate evidence networks (Fig. 2). Since there was no trial investigating any of the treatment regimens from the larger white network with a treatment investigated in the smaller black network, no comparative estimates between treatments of separate networks can be obtained. The analysis was conducted separately for the white and the black network. Tables 2 and 3 shows the relative HRs and 95% credible intervals for each within network comparison.

**Table 2:**
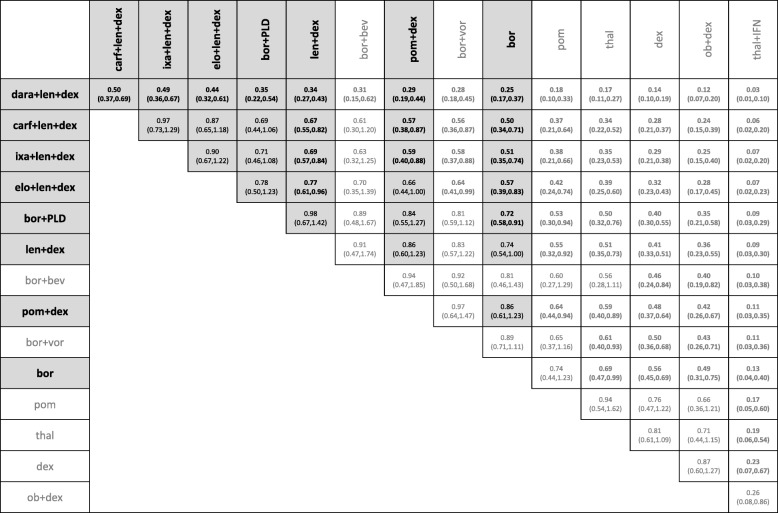
Hazard ratios of progression free survival and 95% credible intervals for within network comparisons based on RCT evidence only for the white network

Licenced treatments and comparisons between those are highlighted in grey. Significant differences on the 95% credible level are in bold

*bev* bevacizumab, *bor* bortezomib, *carf* carfilzomib, *cyc* cyclophosphamide, *dara* daratumumab, *dex* dexamethasone, *elo* elozumatab, *IFN* interferon alpha, *ixa* ixazomib, *len* lenalidomide, *ob* oblimersen, *pan* panobinostat, *peri* perifosine, *PLD* pegylated liposomal doxorubicin, *pom* pomalidomide, *sil* silituximab, *thal* thalidomide, *vor* vorinostat

**Table 3:**
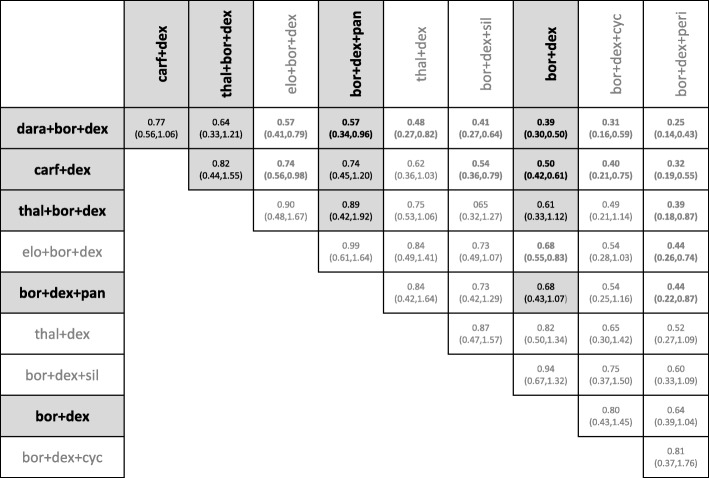
Hazard ratios of progression free survival and 95% credible intervals for within network comparisons based on RCT evidence only for the black network

Licenced treatments and comparisons between those are highlighted in grey. Significant differences on the 95% credible level are in bold

*bev* bevacizumab, *bor* bortezomib, *carf* carfilzomib, *cyc* cyclophosphamide, *dara* daratumumab, *dex* dexamethasone, *elo* elozumatab, *IFN* interferon alpha, *ixa* ixazomib, *len* lenalidomide, *ob* oblimersen, *pan* panobinostat, *peri* perifosine, *PLD* pegylated liposomal doxorubicin, *pom* pomalidomide, *sil* silituximab, *thal* thalidomide, *vor* vorinostat

**Fig. 3:**
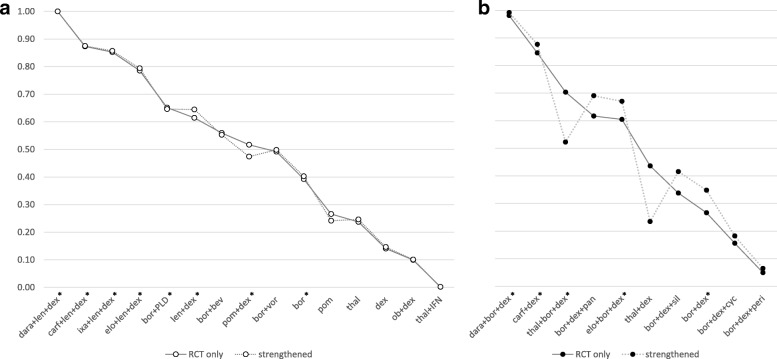
SUCRA score for within network comparisons based on RCT evidence only (solid line) and RCT evidence including matches to strengthen within network evidence (dotted line) ** for **a** the white and **b** the black network. *Interventions with a licence in Europe. ** includes match 1 (Table 5) for white and matches 2 and 3 (Table 5) for black network. bev = bevacizumab; bor = bortezomib; carf = carfilzomib; cyc = cyclophosphamide; dara = daratumumab; dex = dexamethasone; elo = elozumatab; IFN = interferon alpha; ixa = ixazomib; len = lenalidomide; ob = oblimersen; pan = panobinostat; peri = perifosine; PLD = pegylated liposomal doxorubicin; pom = pomalidomide; sil = silituximab; thal = thalidomide; vor = vorinostat

The SUCRA score shown as the solid line in Fig. 3 provides an additional summary statistic of each treatment’s overall ranking. Rankograms showing the probability of each intervention to be ranked best, second best etc. are shown in Additional file 6.

Dara+len + dex was estimated to be the best treatment in the white network with respect to PFS, showing a significant improvement (using the 95% credible intervals) compared to all other treatments in the network. This combination was followed by the other triple combinations (carf, ixa and elo in combination with len + dex), among which no significant differences were observed, which did however, show significant improvements compared to other licensed treatments with the exception of bor + PLD. Lowest efficacy was shown by five unlicensed regimens (pom, thal, dex, ob + dex, thal+IFN), which have shown significantly lower efficacy compared to all licensed interventions (with the exception of bor versus pom). Pom + dex and bor appeared the worst ranked licensed treatments, showing no significant difference to non-licensed regimens bor + bev and bor + vor.

Dara+bor + dex was estimated to be the most efficacious treatment regimen in the black network showing a significant improvement over the remaining treatments except for carf+dex and thal+bor + dex. Three of the other licensed treatments follow (carf+dex, thal+bor + dex and bor + dex + pan), as well as elo + bor + dex with similar efficacy to bor + dex + pan. Lowest efficacy was shown by two unlicensed regimens (bor + dex + peri and bor + dex + cyc). No significant difference was found between the licenced combination bor + dex and any of the unlicensed regimens.

The rank analysis showed an increased uncertainty of bor + bev compared to other treatments in the larger white network (see figure (b) of Additional file 6). This increased uncertainty is likely due to its connection through relatively small trials to the remaining regimens. Similar effects were observed in the black network for a number of regimens.

Due to the disconnected overall network, it is not possible to draw any conclusion on between network comparisons based on RCT evidence alone.

### Analysis of RCTs plus observational studies

After removing trials not reporting the relevant outcome measure or investigating interventions not part of the RCT network, only single armed evidence was left for inclusion. Twelve of these studies provided a full covariate profile and were considered for matching. Table 1 summarises the outcomes and baseline characteristics of these studies. Restricting combinations to matches between trials investigating different treatment regimens, there were a total of 56 possible matches. A distance metric incorporating median age, median number of prior treatment lines, mean baseline stage and proportion of females was calculated for each possible match; results are shown in Table 4.

**Table 4:**
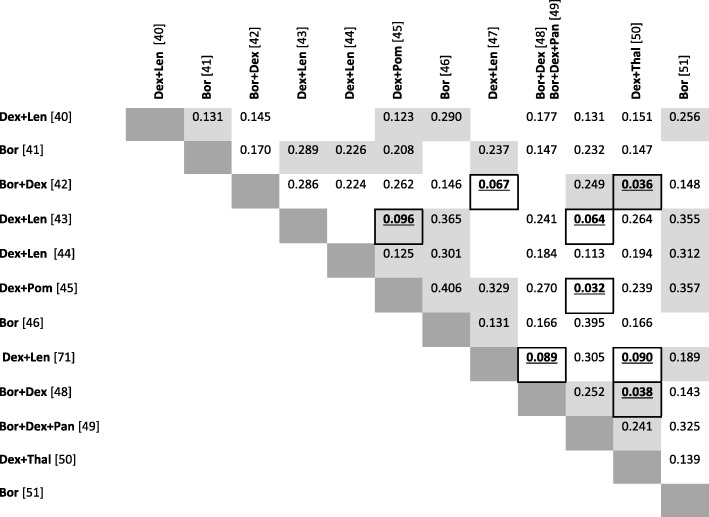
Distance metric between observational studies

Matches of within network pairs are shaded in grey. Pairs explored at a threshold of 0.1 are bold and underlined

*bev* bevacizumab, *bor* bortezomib, *carf* carfilzomib, *cyc* cyclophosphamide, *dara* daratumumab, *dex* dexamethasone, *elo* elozumatab, *IFN* interferon alpha, *ixa* ixazomib, *len* lenalidomide, *ob* oblimersen, *pan* panobinostat, *peri* perifosine, *PLD* pegylated liposomal doxorubicin, *pom* pomalidomide, *sil* silituximab, *thal* thalidomide, *vor* vorinostat

A distance threshold of 0.1 was applied for the base case analysis, and this resulted in the exploration of 14% (*n* = 8) of possible matches, which are underlined and marked in bold in Table 4. Table 5 summarises these 8 matched studies included in the analysis.

**Table 5:** Matches included in base case analysis

ID	Match 1	Match 2	Drug 1	Drug 2	PFS 1^a^	PFS 2^a^	HR^b^
Within white network							
1	Hou et al. [73]	Lacy et al. [74]	dex + len	dex + pom	8.3	4.8	0.58
Within black network							
2	Fukushima et al. [72]	Terpos et al. [78]	bor + dex	dex + thal	16.8	8	0.48
3	Pantani et al. [76]	Terpos et al. [78]	bor + dex	dex + thal	8.7	8	0.92
Connecting both networks							
4	Hou et al. [73]	Richardson et al. [77]	dex + len	bor + dex + pan	8.3	5.4	0.65
5	Lacy et al. [74]	Richardson et al. [77]	dex + pom	bor + dex + pan	4.8	5.4	1.13
6	Oehrlein et al. [75]	Pantani et al. [76]	dex + len	bor + dex	11.6	8.7	0.75
7	Oehrlein et al. [75]	Terpos et al. [78]	dex + len	dex + thal	11.6	8	0.69
8	Fukushima et al. [72]	Oehrlein et al. [75]	bor + dex	dex + len	16.8	11.6	0.69

*bev* bevacizumab, *bor* bortezomib, *carf* carfilzomib, *cyc* cyclophosphamide, *dara* daratumumab, *dex* dexamethasone, *elo* elozumatab, *IFN* interferon alpha, *ixa* ixazomib, *len* lenalidomide, *ob* oblimersen, *pan* panobinostat, *peri* perifosine, *PLD* pegylated liposomal doxorubicin, *pom* pomalidomide, *sil* silituximab, *thal* thalidomide, *vor* vorinostat, *HR* hazard ratio, *PFS* progression free survival

^a^median

^b^estimated from median PFS assuming exponential survival

**Fig. 4:**
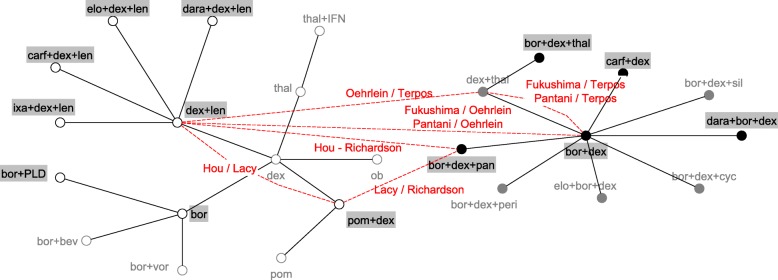
Evidence network including single armed matches: Each node represents a treatment regimen; solid connections between nodes indicate comparative RCT evidence, dotted connections indicate single armed matches. Interventions licensed in Europe are highlighted in grey. bev = bevacizumab; bor = bortezomib; carf = carfilzomib; cyc = cyclophosphamide; dara = daratumumab; dex = dexamethasone; elo = elozumatab; IFN = interferon alpha; ixa = ixazomib; len = lenalidomide; ob = oblimersen; pan = panobinostat; peri = perifosine; PLD = pegylated liposomal doxorubicin; pom = pomalidomide; sil = silituximab; thal = thalidomide; vor = vorinostat

Five studies had no matched pair below the threshold and were not included in the base case [40–44]. Of the eight matches explored, one strengthens the within white network evidence, two the within black network evidence and five matches connect both networks allowing for a comparison between all treatment regimens. The evidence network including these 8 matches is shown in Fig. 4.

We first explored the impact of including matches strengthening the within network evidence (match 1 for white and matches 2 and 3 for black network (Table 5)), matches connecting both networks are explored in a second step.

The grey dotted line in Fig. 3 shows the SUCRA score of the first step. The impact of adding a match connecting len + dex and pom + dex in the white network is minimal indicating that the evidence added does not contradict the RCT evidence. Due to network properties, adding matches for the comparative effect of bor + dex and dex + thal only affects the relative effects of these regimens as well as bor + dex + thal. The ranking shows a decrease in SUCRA score for bor + dex + thal as well as dex + thal; however, one should note that the reordering in the ranking only affects interventions between which no significant difference was observed.

The second step analysed the matches connecting both networks. A model incorporating all five connecting matches was fitted as well as five models investigating each match in turn.

**Fig. 5:**
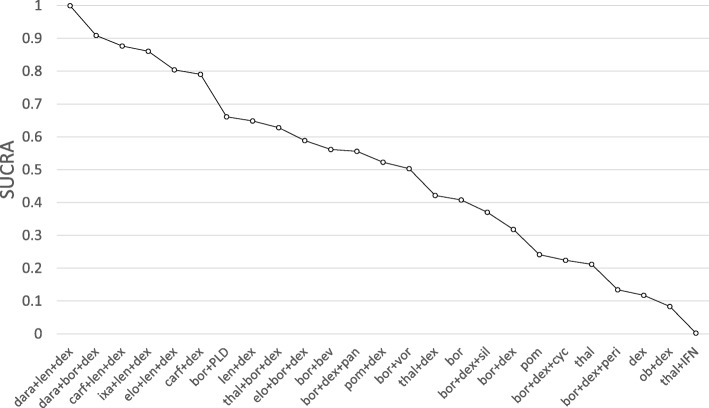
SUCRA scores of analyses connecting separate networks of evidence. Shows ranking of model including all connecting matches simultaneously as well as models investigating each match individually. bev = bevacizumab; bor = bortezomib; carf = carfilzomib; cyc = cyclophosphamide; dara = daratumumab; dex = dexamethasone; elo = elozumatab; IFN = interferon alpha; ixa = ixazomib; len = lenalidomide; ob = oblimersen; pan = panobinostat; peri = perifosine; PLD = pegylated liposomal doxorubicin; pom = pomalidomide; sil = silituximab; thal = thalidomide; vor = vorinostat

The relative HRs and 95% credible intervals of comparisons between treatments licensed in Europe based on the model incorporating all five connections is displayed in Table 6, the SUCRA plot is shown in Fig. 5. All pairwise comparisons including those of unlicensed treatments can be found as Additional file 7.

**Table 6:** Pairwise hazard ratios and 95% credible intervals of interventions licensed in Europe based on RCT evidence as well as all 5 matches connecting the separate networks satisfying the similarity threshold

	dara+bor + dex	carf+len + dex	ixa + len + dex	elo + len + dex	carf+dex	bor + PLD	len + dex	thal+bor + dex	bor + dex + pan	pom + dex	bor	bor + dex
dara+len + dex	*0.56 (0.35,0.87)*	*0.51 (0.37,0.70)*	*0.49 (0.36,0.67)*	*0.44 (0.32,0.62)*	*0.43 (0.28,0.65)*	*0.35 (0.22,0.53)*	*0.34 (0.27,0.43)*	*0.33 (0.18,0.62)*	*0.30 (0.20,0.44)*	*0.29 (0.19,0.42)*	*0.25 (0.17,0.37)*	*0.22 (0.15,0.32)*
dara+bor + dex		0.91 (0.58,1.42)	0.88 (0.56,1.36)	0.80 (0.50, 1.24)	0.77 (0.56,1.06)	0.62 (0.37,1.06)	*0.61 (0.40,0.90)*	0.60 (0.33,1.08)	*0.54 (0.39,0.74)*	*0.52 (0.32,0.83)*	*0.45 (0.28,0.72)*	*0.39 (0.30,0.50)*
carf+len + dex			0.97 (0.73,1.27)	0.87 (0.64,1.18)	0.85 (0.56,1.29)	0.68 (0.44,1.04)	*0.67 (0.55,0.82)*	0.66 (0.35,1.20)	*0.59 (0.41,0.85)*	*0.57 (0.39,0.83)*	*0.49 (0.34,0.71)*	*0.43 (0.29,0.61)*
ixa + len + dex				0.90 (0.67,1.22)	0.88 (0.58,1.30)	0.70 (0.47,1.07)	*0.69 (0.57,0.84)*	0.68 (0.37,1.24)	*0.61 (0.43,0.87)*	*0.59 (0.41,0.85)*	*0.51 (0.36,0.72)*	*0.44 (0.31,0.63)*
elo + len + dex					0.97 (0.63,1.50)	0.78 (0.51,1.22)	*0.77 (0.61,0.96)*	0.75 (0.41,1.39)	*0.68 (0.47,0.99)*	*0.65 (0.44,0.96)*	*0.56 (0.39,0.82)*	*0.49 (0.33,0.72)*
carf+dex						0.80 (0.49,1.32)	0.79 (0.55,1.13)	0.78 (0.44,1.37)	*0.70 (0.53,0.91)*	0.67 (0.43,1.06)	*0.58 (0.37,0.79)*	*0.50 (0.42,0.60)*
bor + PLD							0.98 (0.68,1.42)	0.97 (0.49,1.89)	0.87 (0.55,1.37)	0.83 (0.56,1.24)	*0.72 (0.58,0.91)*	*0.63 (0.39,0.99)*
len + dex								0.98 (0.55,1.74)	0.88 (0.66,1.20)	0.85 (0.62,1.16)	*0.74 (0.55,0.99)*	*0.64 (0.47,0.86)*
thal+bor + dex									0.90 (0.51,1.60)	0.86 (0.46,1.63)	0.75 (0.40,1.43)	0.65 (0.38,1.12)
bor + dex + pan										0.96 (0.66,1.42)	0.83 (0.55,1.23)	*0.72 (0.60,0.88)*
pom + dex											0.87 (0.62,1.20)	0.75 (0.50,1.11)
bor												0.87 (0.58,1.30)

*bev* bevacizumab, *bor* bortezomib, *carf* carfilzomib, *cyc* cyclophosphamide, *dara* daratumumab, *dex* dexamethasone, *elo* elozumatab, *IFN* interferon alpha, *ixa* ixazomib, *len* lenalidomide, *ob* oblimersen, *pan* panobinostat, *peri* perifosine, *PLD* pegylated liposomal doxorubicin, *pom* pomalidomide, *sil* silituximab, *thal* thalidomide, *vor* vorinostat

Significant results on the 95% level are marked in italic

Dara+len + dex is estimated the most efficacious treatment showing significant improvement compared to all other licensed interventions. Dara+bor + dex, carf+len + dex, ixa + len + dex, elo + len + dex and carf+dex follow, showing no significant differences between each other. With the exception of carf+dex, these treatments show superiority over the remaining strategies, with the exception of thal+bor + dex. Pom + dex, bor and bor + dex show the least efficacy of all licensed treatments and no significant differences among each other. In the ranking of all investigated interventions, eight of the licensed interventions show the highest efficacy and none of the unlicensed strategies show a significant improvement over any of the licensed strategies. However, some of the unlicensed strategies such as elo + bor + dex or bor + bev appear to have similar effects to many licensed treatment pathways.

The SUCRA score of licensed treatments of all scenarios individually are shown as Additional file 8. While the relative ranking of treatments within each network remains unchanged, considerable variation on inter-network comparisons is observed. However, dara+len + dex remains best or second-best treatment strategy in all scenarios (dara+len + dex is exceeded by dara+bor + dex in match 8). Dara+bor + dex also remains in the top 5 treatment strategies in all scenarios. The triple combinations with len + dex are also ranked in the top 5, except for scenario 5, where carf+dex, and triple combinations with bor + dex are ranked higher.

To validate our analysis we compared our outcomes with those of existing inter network comparisons (see outcomes summarised in Table 7). While no gold standard exists, two analyses based on individual patient level data have estimated the relative effect between bor + dex and bor with respect to PFS [29, 39] representing the currently best available evidence. Two recent NMAs made assumptions based on clinical opinion on the same comparison. None of the studies significantly favours either strategy. The two NMAs assume equal efficacy of both interventions, while both individual patient level data studies show a tendency favouring bor + dex. While the point estimate in our study favours bor, there is a large overlap in the confidence intervals. Further, our analysis shows the highest variance in the estimate, which is appropriate given the risk of matching single armed studies, especially based on summary data.

**Table 7:** Estimated Mean Hazard Ratio and 95% confidence interval of bor + dex versus bor comparison in different studies

	HR bor + dex versus bor
Dimopoulous 2010 [39]	0.73 (0.52,1.03)*
Dimopoulous 2014 [29]	0.60 (0.35,1.01)
Botta [31]	1 (−)
Van Beurden-Tan [3]	1 (−)
Matched analysis	1.15 (0.77,1.74)

*bor* bortezomib, *dex* dexamethasone, *HR* hazard ratio

*confidence interval estimated using *p*-value

The sensitivity of choice of threshold has been evaluated for this application previously [38]. The analysis investigated the trade-off between strict thresholds, which would reduce the number of matches explored and therefore potentially underestimate the uncertainty, and high thresholds, which may include matches of trials with very different patient populations. A threshold of 0.1 appeared to explore a reasonable level of uncertainty. In addition, we analysed the similarity between arms within RCT trials using the same metric. The analysis was restricted to those studies which report a full covariate profile for each arm. Results indicate that different arms of the same study have an average distance of 0.01 ranging from 0.00 to 0.03. This indicates that a threshold of 0.1 allows for the inclusion of matched pairs which are less similar compared to different arms within an RCT. Only match 5 would be considered if a threshold in line with the distances observed within RCT studies was applied.

## Discussion

The purpose of this analysis was to illustrate how observational data can be used to link otherwise disconnected evidence networks and aid the estimation of relative effectiveness between treatments, which would not be possible otherwise, while acknowledging and communicating the additional uncertainty associated with such an approach.

Clinical research into pharmacological interventions for RRMM is a vast and growing field. The large number of treatment regimens explored over the years form a complex evidence structure, for which standard methods for evidence synthesis fail to produce estimates of relative efficacy between all treatments.

Previous analyses have attempted to solve the problem of disconnected networks by grouping regimens and assuming equal efficacy for each group [3, 31]. While this approach allows for the estimation of relative effects across the entire evidence base, the uncertainty associated with the assumption is not incorporated. Since grouping is done with the aim of connecting disconnected networks, there is likely no clinical evidence supporting equal efficacy for these interventions. Communicating results without incorporating additional layers of uncertainty bares the risk of overconfident interpretation of results. Two studies have used individual patient level data to obtain the relative effects between bor and bor + dex [29, 39]. While such analysis can only account for observed covariates, such analyses provide the best available evidence in the absence of RCT evidence.

In the absence of individual patient level data, we propose the use of study level data to match single armed trials to fill the gap in RCT evidence.

Optimal matching based on summary data is not new, see for example the work of Rosenbaum [45]; Jaff et al. provides a recent example of optimal matching in peripheral artery disease [46]. Since matching based on study level data is prone to bias, capturing uncertainty is key. The selection of one optimal match may underestimate the uncertainty associated with the methodology. We therefore explored the space of possible matches and the impact different matches have on the results. While general agreement between scenarios can be observed (higher ranked treatment in either network remain among the higher ranked treatments overall), considerable variation in the rank distribution is observed nevertheless. This variation is translated into an increased variance of estimates of relative effect between both networks.

The focus of this article is the methodology applied; the HRs reported in the results should not be interpreted as hard point estimates. Our analysis indicates that triple combinations with daratumumab as well as triple combinations with len + dex provide the highest efficacy relative to remaining treatments, with respect to PFS. Thal+IFN shows least effects throughout all scenarios.

### Limitations

Median PFS was used to compare all treatment regimens, reflecting the outcome most widely reported across studies. Analyses of other outcomes, such as overall survival may have produced different results and further research should consider additional outcomes of interest.

Observational studies identified in the initial search varied in methods, from study design through to outcome reporting, and ultimately only 12 studies were considered to supplement RCT evidence. Matching based on study level information is prone to bias, making appropriate capturing of uncertainty highly important. There is no guidance on how similar is “similar enough”. Using a low threshold may result in the underestimation of uncertainty, while a high threshold may result in matching trials too dissimilar to provide useful comparisons. While a threshold of 0.1 appears to provide a reasonable exploration of the associated uncertainty, it is worth noting that differences within RCTs are much lower. Using the same approach, we have calculated the distance between arms within RCTs included in this analysis (where data was available) and the maximum distance observed was 0.03.

We only allowed for matching observational studies with each other to avoid interfering with the RCTs (either by duplicating an arm or inserting an extra arm). Alternative to matching single armed observational evidence with each other, we could have matched observational studies directly to RCTs or connect RCTs with each other [7, 47]. The distance metric indicates similar differences for all approaches (average distance 0.17 (range 0.01–0.48) within RCTs, 0.19 (range 0.02, 0.47) RCT to observational, 0.20 (range 0.03, 0.47) within observational); however, considering a larger space of matches may improve the analysis of variation.

## Conclusions

Where RCT evidence alone results in a disconnected evidence structure, additional information can often be obtained from observational evidence. This paper presents a novel approach to establish a ranking of available treatment regimens in disconnected evidence networks through the incorporation of observational studies, taking into account the associated uncertainty of matching single armed trials. Applying this method to RRMM, we present the relative efficacy of available treatment regimens, which is not possible to obtain using standard methods.

## Additional files

Additional file 1: Search strategy. Details the search strategy applied for the systematic review. (PDF 1100 kb)

Additional file 2: WinBUGs code. Contains WinBUGs code and input data for the analysis. (PDF 1103 kb)

Additional file 3: Numerical example. Shows a numerical example illustrating the calculation of the distance measure between single armed studies. (PDF 1101 kb)

Additional file 4: Reason for exclusion. Details the reason for excluding studies in the quantitative analysis. (PDF 1102 kb)

Additional file 5: Quality assessment. Shows the quality assessment of RCTs and observational studies included in the analysis. (PDF 1103 kb)

Additional file 6: Rankogram. Shows the rankograms for the white and the black network of the RCT only analysis. (PDF 1103 kb)

Additional file 7: All pairwise comparisons. Table containing hazard ratios and 95% credible intervals of all pairwise comparisons in the combined analysis. (PDF 1100 kb)

Additional file 8: SUCRA all scenarios. SUCRA ranking score of all licensed treatments of individual matches connecting both networks as well as the base case scenario containing all matches. (PDF 1100 kb)

## Abbreviations

ADS: Activity daily score
bev: Bevacizumab
bor: Bortezomib
carf: Carfilzomib
CCS: Charlsen’s comorbidity score
cyc: Cyclophosphamide
dex: Dexamethasone
elo: Elozumatab
HR: Hazard ratios
IFN: Interferon alpha
ixa: Ixazomib
len: Lenalidomide
MM: Multiple myeloma
NMA: Network meta analysis
NOS: Newcastle Ottowa Scale
ob: Oblimersen
OS: Overall survival
pan: Panobinostat
peri: Perifosine
PFS: Progression free survival
PLD: Pegylated liposomal doxorubicin
RCTs: Randomised control trials
RRMM: Relapsed and refractory multiple myeloma
sil: Silituximab
SUCRA: Surface under the cumulative ranking curve
thal: Thalidomide
TTP: Time to progression
vor: Vorinostat
