# Supplementary material for: The use of single armed observational data to closing the gap in otherwise disconnected evidence networks: a network meta-analysis in multiple myeloma
Source: BMC Med Res Methodol. 2018 Jun 28;18:66. doi: 10.1186/s12874-018-0509-7 (PMC6022299; doi:10.1186/s12874-018-0509-7)
Supplement: Supplementary file 1 — Search strategy. Details the search strategy applied for the systematic review. (PDF 1100 kb) [file 12874_2018_509_MOESM1_ESM.pdf]

Search Strategy

1) Embase
    a. RCT
        1. (relapsed adj2 refractory adj5 "multiple myeloma*").ti,ab.
        2. Clinical trial/
        3. Randomized controlled trial/
        4. Randomization/
        5. Single blind procedure/
        6. Double blind procedure/
        7. Crossover procedure/
        8. Placebo/
        9. Randomi?ed controlled trial$.tw.
        10. Rct.tw.
        11. Random allocation.tw.
        12. Randomly allocated.tw.
        13. Allocated randomly.tw.
        14. (allocated adj2 random).tw.
        15. Single blind$.tw.
        16. Double blind$.tw.
        17. Placebo$.tw.
        18. ((treble or triple) adj blind$).tw.
        19. Prospective study/
        20. or/2-19
        21. Case study/
        22. Case report.tw.
        23. Abstract report/ or letter/
        24. 21 or 22 or 23
        25. 20 not 24
        26. 1 and 25
    b. Observational
        1. (relapsed adj2 refractory adj5 "multiple myeloma*").ti,ab.
        2. Clinical study/
        3. Case control study.mp. or exp case control study/
        4. Family study/
        5. Longitudinal study/
        6. Retrospective study/
        7. Prospective study/
        8. Randomized controlled trials/
        9. 7 not 8
        10. Cohort analysis/
        11. (Cohort adj (study or studies)).mp.
        12. (Case control adj (study or studies)).tw.
        13. (follow up adj (study or studies)).tw.
        14. (observational adj (study or studies)).tw.
        15. (epidemiologic$ adj (study or studies)).tw.
        16. (cross sectional adj (study or studies)).tw.
        17. or/2-6,9-16
        18. 1 and 17
2) Medline
    a. RCT
        1. exp Multiple Myeloma/
        2. (relapsed adj2 refractory adj5 "multiple myeloma*").ti,ab.
        3. (relapsed and refractory).ti,ab.
        4. 1 and 3
        5. 2 or 4
        6. Randomized Controlled Trials as Topic/
        7. randomized controlled trial/
        8. Random Allocation/
        9. Double Blind Method/
        10. Single Blind Method/

11. clinical trial/
12. clinical trial, phase i.pt.
13. clinical trial, phase ii.pt.
14. clinical trial, phase iii.pt.
15. clinical trial, phase iv.pt.
16. controlled clinical trial.pt.
17. randomized controlled trial.pt.
18. multicenter study.pt.
19. clinical trial.pt.
20. exp Clinical Trials as topic/
21. (clinical adj trial$).tw.
22. exp Clinical Trials as topic/
23. (clinical adj trial$).tw.
24. ((singl$ or doubl$ or treb$ or tripl$) adj (blind$3 or mask$3)).tw.
25. PLACEBOS/
26. placebo$.tw.
27. randomly allocated.tw.
28. (allocated adj2 random$).tw.
29. or/6-28
30. case report.tw.
31. letter/
32. historical article/
33. or/30-32
34. 29 not 33
35. 5 and 34

b. Observational

1. exp Multiple Myeloma/
2. (relapsed adj2 refractory adj5 "multiple myeloma*").ti,ab.
3. (relapsed and refractory).ti,ab.
4. 1 and 3
5. 2 or 4
6. Epidemiologic studies/
7. exp case control studies/
8. exp cohort studies/
9. Case control.tw.
10. (cohort adj (study or studies)).tw.
11. Cohort analy$.tw.
12. (Follow up adj (study or studies)).tw.
13. (observational adj (study or studies)).tw.
14. Longitudinal.tw.
15. Retrospective.tw.
16. Cross sectional.tw.
17. Cross-sectional studies/
18. or/6-17
19. 5 and 18

3) Cochrane Library

ID	Search
#1	MeSH descriptor: [Multiple Myeloma] explode all trees
#2	"multiple myeloma" and relapsed and refractory:ti,ab,kw (Word variations have been searched)
#3	relapsed and refractory
#4	#1 and #3
#5	#2 or #4
