# Supplementary material for: The use of single armed observational data to closing the gap in otherwise disconnected evidence networks: a network meta-analysis in multiple myeloma
Source: BMC Med Res Methodol. 2018 Jun 28;18:66. doi: 10.1186/s12874-018-0509-7 (PMC6022299; doi:10.1186/s12874-018-0509-7)
Supplement: Supplementary file 2 — WinBUGs code. Contains WinBUGs code and input data for the analysis. (PDF 1103 kb) [file 12874_2018_509_MOESM2_ESM.pdf]

Winbugs Code

```
model{

for (i in 1:ns){                                          ### loop through number of studies ns
        delta[i,bi[i]] <- 0
        mu[i] ~ dnorm(0,0.01)
        for (k in 1:na[i]){
                r[i,k] ~ dbin(p[i,k],n[i,k])              ### binomial likelihood function
                p[i,k] <- 1- exp(-w[i,k]*exp(loga[i,k]))  ### exponential survival to estimate log hazard
                loga[i,k] <- mu[i] + delta[i,t[i,k]]      ### estimation of log hazard ratio
                }
        for (k in 2:na[i]){
                delta[i,si[i,k]] <- d[si[i,k]] - d[bi[i]] ### fixed effects
                }
        }
        d[1] <- 0
        for (k in 2:nt){d[k] ~ dnorm(0,0.01) }
        for (c in 1:nt) {
                for (k in 1:nt) {
                        IC[c,k] <- d[c]-d[k]}}            ### estimation of pairwise comparisons
}
```

Example Input data (white network)

```
WINdata<-list("r"=r, "n"=n, "t"=t, "na" = na, "nt" = nt, "ns" = ns, "bi" = bi, "si" = si, "w"=w)
```

$r number of patients alive at time w

```
      N1  N2
 [1,]  63 186
 [2,] 161 162
 [3,]  26  24
 [4,] 160 158
 [5,] 168 166
 [6,]   6   8
 [7,]  88  88
 [8,]  88  88
 [9,] 198 198
[10,]  56  54
[11,]  76 151
[12,]  57  55
[13,] 162 160
[14,] 182 180
[15,]  29  28
[16,] 142 143
```

$n number of patients in each study arm

```
      N1  N2
 [1,] 126 373
 [2,] 322 324
 [3,]  53  49
 [4,] 320 317
 [5,] 336 333
```

```
 [6,]  12  16
 [7,] 175 176
 [8,] 176 177
 [9,] 396 396
[10,] 113 108
[11,] 153 302
[12,] 114 110
[13,] 325 321
[14,] 363 360
[15,]  58  57
[16,] 283 286
```

$t treatment index of treatment given in each study arm

```
     T1 T2
 [1,]  1  4
 [2,]  2  6
 [3,]  2  7
 [4,]  2  8
 [5,]  1  2
 [6,]  4  9
 [7,]  1  3
 [8,]  1  3
 [9,]  3 10
[10,]  5 11
[11,]  1  5
[12,]  1 12
[13,]  3 13
[14,]  3 14
[15,]  3 14
[16,]  3 15
```

$na number of arms in each study

```
 [1] 2 2 2 2 2 2 2 2 2 2 2 2 2 2 2 2
```

$nt number of treatments

```
[1] 15
```

$ns number of studies

```
[1] 16
```

$bi baseline treatment

```
 [1] 1 2 2 2 1 4 1 1 3 5 1 1 3 3 3 3
```

$si comparator treatment

```
     [,1] [,2]
 [1,]   NA    4
 [2,]   NA    6
 [3,]   NA    7
 [4,]   NA    8
 [5,]   NA    2
 [6,]   NA    9
 [7,]   NA    3
 [8,]   NA    3
 [9,]   NA   10
[10,]   NA   11
[11,]   NA    5
```

```
[12,]   NA   12
[13,]   NA   13
[14,]   NA   14
[15,]   NA   14
[16,]   NA   15
```

$w time point of survival measure

```
      medPFS1 medPFS2
 [1,]    6.00    7.40
 [2,]    6.50    9.00
 [3,]    5.10    6.20
 [4,]    6.83    7.63
 [5,]    3.49    6.22
 [6,]    7.90    1.50
 [7,]    4.70   11.30
 [8,]    4.70   11.10
 [9,]   17.60   26.30
[10,]    4.20    2.70
[11,]    1.90    4.00
[12,]    3.55    3.09
[13,]   14.90   19.40
[14,]   14.70   20.60
[15,]    4.00    6.70
[16,]   18.40   54.10
```
