# Supplementary material for: The use of single armed observational data to closing the gap in otherwise disconnected evidence networks: a network meta-analysis in multiple myeloma
Source: BMC Med Res Methodol. 2018 Jun 28;18:66. doi: 10.1186/s12874-018-0509-7 (PMC6022299; doi:10.1186/s12874-018-0509-7)
Supplement: Supplementary file 3 — Numerical example. Shows a numerical example illustrating the calculation of the distance measure between single armed studies. (PDF 1101 kb) [file 12874_2018_509_MOESM3_ESM.pdf]

**Numerical example illustrating the calculation of the distance measure between single armed studies**

1. Data example :

| trial | treatment | prior lines (median) | age (median) | baseline stage (mean) | female (%) |
|---|---|---|---|---|---|
| Avet-Loiseau et al. [54] | dex+len | 3 | 65 | 1.7 | 44 |
| Fukushima et al. [56] | bor+dex | 2 | 69 | 2.1 | 41 |

2. Normalise outcomes on baseline characteristics using the assumed range of possible values and calculate absolute differences

| Trial | treatment | prior lines | age | baseline stage | female (%) |
|---|---|---|---|---|---|
| Assumed range | - | 1-4 | 20-100 | 1-3 | 0-100 |
| weight | - | 4 | 3 | 2 | 1 |
| Avet-Loiseau et al. [54] | dex+len | 0.75 | 0.56 | 0.57 | 0.44 |
| Fukushima et al. [56] | bor+dex | 0.50 | 0.61 | 0.70 | 0.41 |
| Δ individual characteristics | | 0.250 | 0.050 | 0.133 | 0.030 |

3. Calculated the weighted average as the distance measure between the two trials

$$\Delta_{tot} = \frac{4 \cdot 0.250 + 3 \cdot 0.050 + 2 \cdot 0.133 + 1 \cdot 0.030}{10} = 0.145$$
