# Supplementary material for: The use of single armed observational data to closing the gap in otherwise disconnected evidence networks: a network meta-analysis in multiple myeloma
Source: BMC Med Res Methodol. 2018 Jun 28;18:66. doi: 10.1186/s12874-018-0509-7 (PMC6022299; doi:10.1186/s12874-018-0509-7)
Supplement: Supplementary file 4 — Reason for exclusion. Details the reason for excluding studies in the quantitative analysis. (PDF 1102 kb) [file 12874_2018_509_MOESM4_ESM.pdf]

Excluded Studies

1. RCTs

Study	Year	Reason for exclusion
**Dimopoulos et al. [1]**	2013	Does not report outcome of interest
**Gertz et al. [2]**	1995	Investigates different doses of the same intervention.
**Jagannath et al. [3]**	2004	Investigates different doses of the same intervention.
**Moreau et al. [4]**	2012	Investigates different doses of the same intervention.<br>Does not report outcome of interest
**Moreau et al. [5]**	2008	Investigates different doses of the same intervention.<br>Does not report outcome of interest
**Richardson et al. [6]**	2006	Investigates different doses of the same intervention.
**Schey et al. [7]**	2013	Investigates different doses of the same intervention.
**Yakoub-Agha et al. [8]**	2011	Investigates different doses of the same intervention.
**Zonder et al. [9]**	2015	Does not report outcome of interest.
**Hajek et al. [10]**	2017	Not connected to network

2. Non-RCTs

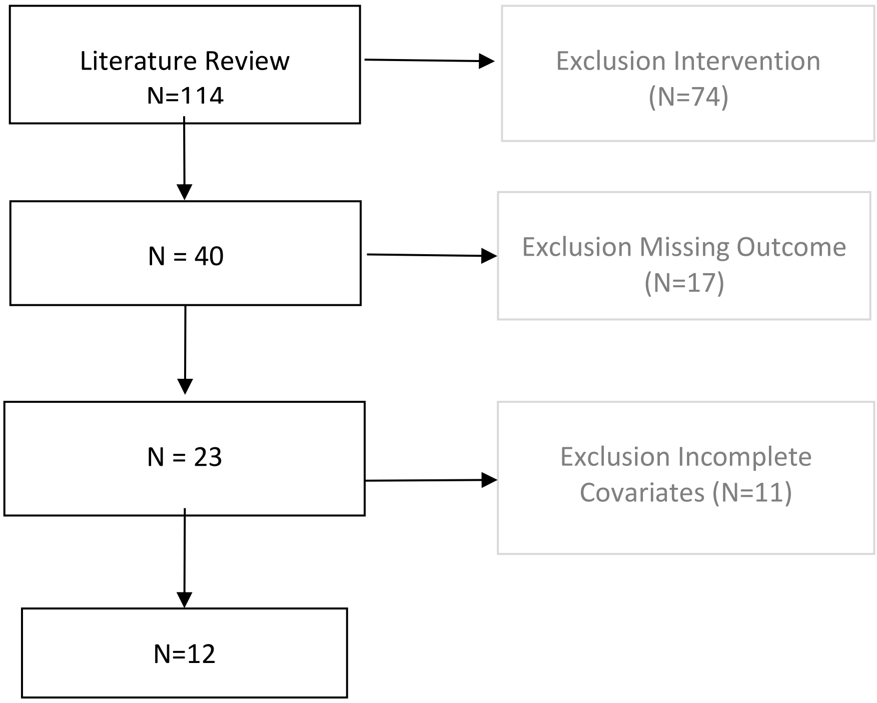

1. Dimopoulos, M.A., et al., *Phase II study of bortezomib–dexamethasone alone or with added cyclophosphamide or lenalidomide for sub-optimal response as second-line treatment for patients with multiple myeloma.* Haematologica, 2013. **98**(8): p. 1264-1272.

2. Gertz, M.A., et al., *Phase III study comparing vincristine, doxorubicin (Adriamycin), and dexamethasone (VAD) chemotherapy with VAD plus recombinant interferon alfa-2 in refractory or relapsed multiple myeloma. An Eastern Cooperative Oncology Group study.* American journal of clinical oncology, 1995. **18**(6): p. 475-480.

3. Jagannath, S., et al., *A phase 2 study of two doses of bortezomib in relapsed or refractory myeloma.* British journal of haematology, 2004. **127**(2): p. 165-172.

4. Moreau, P., et al., *A randomized phase II study of elotuzumab with lenalidomide and low-dose dexamethasone in patients with relapsed/refractory multiple myeloma*. 2012, American Society of Clinical Oncology.

5. Moreau, P., et al., *Prospective comparison of subcutaneous versus intravenous administration of bortezomib in patients with multiple myeloma.* haematologica, 2008. **93**(12): p. 1908-1911.

6. Richardson, P.G., et al., *A randomized phase 2 study of lenalidomide therapy for patients with relapsed or relapsed and refractory multiple myeloma.* Blood, 2006. **108**(10): p. 3458-3464.

7. Yong, K.L., et al., *Identifying an optimally effective but tolerable dose of bendamustine in combination with thalidomide and dexamethasone in patients with relapsed or refractory multiple myeloma*. 2013, Am Soc Hematology.

8. Yakoub-Agha, I., et al., *Low-dose vs. high-dose thalidomide for advanced multiple myeloma: a prospective trial from the Intergroupe Francophone du Myélome.* European journal of haematology, 2012. **88**(3): p. 249-259.

9. Zonder, J.A., et al., *Phase 2 study of carfilzomib (CFZ) with or without filanesib (FIL) in patients with advanced multiple myeloma (MM)*. 2015, Am Soc Hematology.

10. Hájek, R., et al., *A randomized phase III study of carfilzomib vs low-dose corticosteroids with optional cyclophosphamide in relapsed and refractory multiple myeloma (FOCUS).* Leukemia, 2017. **31**(1): p. 107.
