# Supplementary material for: The use of single armed observational data to closing the gap in otherwise disconnected evidence networks: a network meta-analysis in multiple myeloma
Source: BMC Med Res Methodol. 2018 Jun 28;18:66. doi: 10.1186/s12874-018-0509-7 (PMC6022299; doi:10.1186/s12874-018-0509-7)
Supplement: Supplementary file 5 — Quality assessment. Shows the quality assessment of RCTs and observational studies included in the analysis. (PDF 1103 kb) [file 12874_2018_509_MOESM5_ESM.pdf]

Study Quality

(A) RCT studies

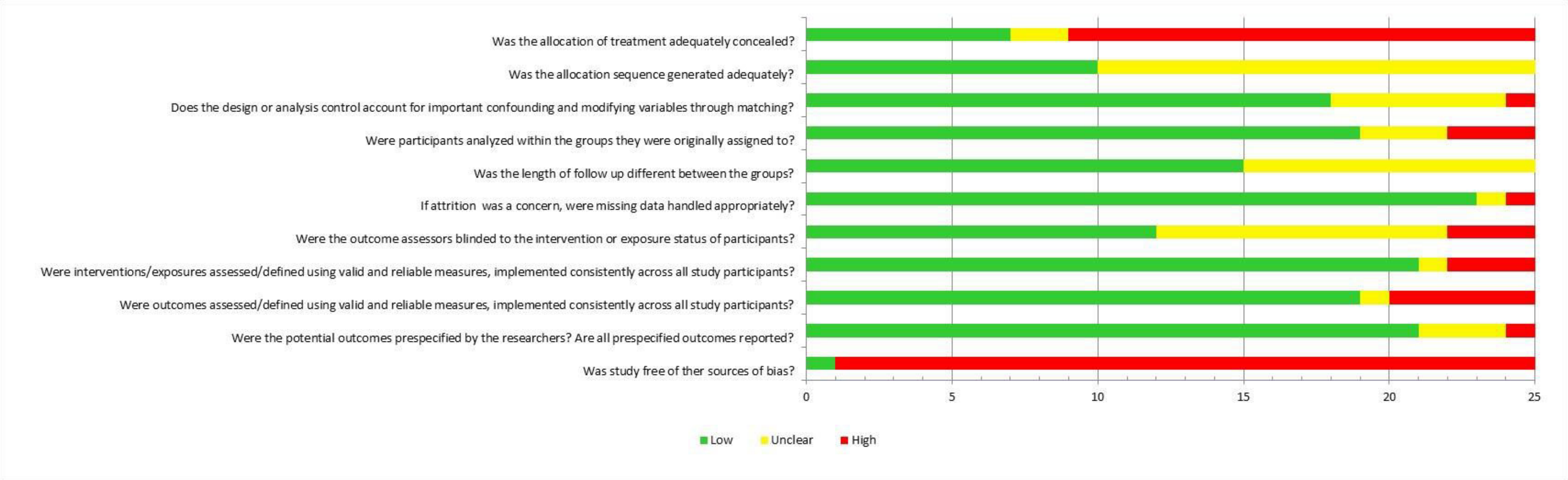

Figure 1: Cochrane Risk of Bias Assessment of Randomised Controlled Trials included in the Analysis

(B) Non-RCT studies

The quality of non-RCT studies was assessed using the Newcastle-Ottawa-Scale :

**Trial name**	**selection**	**outcome**	**Agreed total stars / 6**
**Avet-Loiseau et al. [1]**	**	***	**5**
**Chang et al. [2]**	*	***	**4**
**Fukushima et al. [3]**	*	***	**4**
**Hou et al. [4]**	**	***	**5**
**Kneppers et al. [5]**	**	***	**5**
**Lacy et al. [6]**	**	***	**5**
**Moore et al. [7]**	*	***	**4**
**Oehrlein et al. [8]**	**	***	**5**
**Pantani et al. [9]**	*	***	**4**
**Richardson et al. [10]**	**	***	**5**
**Terpos et al. [11]**	**	***	**5**
**Walter-Croneck et al. [12]**	**	***	**5**

1. Avet-Loiseau, H., et al., *Impact of high-risk cytogenetics and prior therapy on outcomes in patients with advanced relapsed or refractory multiple myeloma treated with lenalidomide plus dexamethasone.* Leukemia, 2010. **24**(3): p. 623.
2. Chang, H., et al., *Bortezomib therapy response is independent of cytogenetic abnormalities in relapsed/refractory multiple myeloma.* Leukemia research, 2007. **31**(6): p. 779-782.
3. Fukushima, T., et al., *Efficacy and safety of bortezomib plus dexamethasone therapy for refractory or relapsed multiple myeloma: once-weekly administration of bortezomib may reduce the incidence of gastrointestinal adverse events.* Anticancer research, 2011. **31**(6): p. 2297-2302.
4. Hou, J., et al., *A multicenter, open-label, phase 2 study of lenalidomide plus low-dose dexamethasone in Chinese patients with relapsed/refractory multiple myeloma: the MM-021 trial.* Journal of hematology & oncology, 2013. **6**(1): p. 1.
5. Kneppers, E., et al., *Analysis of efficacy and prognostic factors of lenalidomide treatment as part of a Dutch compassionate use program.* Clinical Lymphoma Myeloma and Leukemia, 2010. **10**(2): p. 138-143.
6. Lacy, M., et al., *Pomalidomide (CC4047) plus low dose dexamethasone (Pom/dex) is active and well tolerated in lenalidomide refractory multiple myeloma (MM).* Leukemia, 2010. **24**(11): p. 1934-1939.
7. Moore, S., et al., *Weekly intravenous bortezomib is effective and well tolerated in relapsed/refractory myeloma.* European journal of haematology, 2013. **90**(5): p. 420-425.
8. Oehrlein, K., et al., *Successful treatment of patients with multiple myeloma and impaired renal function with lenalidomide: results of 4 German centers.* Clinical Lymphoma Myeloma and Leukemia, 2012. **12**(3): p. 191-196.
9. Pantani, L., et al., *Bortezomib and dexamethasone as salvage therapy in patients with relapsed/refractory multiple myeloma: analysis of long-term clinical outcomes.* Annals of hematology, 2014. **93**(1): p. 123-128.
10. Richardson, P.G., et al., *PANORAMA 2: panobinostat in combination with bortezomib and dexamethasone in patients with relapsed and bortezomib-refractory myeloma.* Blood, 2013. **122**(14): p. 2331-2337.
11. Terpos, E., et al., *The combination of intermediate doses of thalidomide with dexamethasone is an effective treatment for patients with refractory/relapsed multiple myeloma and normalizes abnormal bone remodeling, through the reduction of sRANKL/osteoprotegerin ratio.* Leukemia, 2005. **19**(11): p. 1969-1976.
12. Walter-Croneck, A., et al., *Case-adjusted bortezomib-based strategy in routine therapy of relapsed/refractory multiple myeloma shown to be highly effective—A report by Polish Myeloma Study Group.* Leukemia research, 2014. **38**(7): p. 788-794.
