# Supplementary material for: The use of single armed observational data to closing the gap in otherwise disconnected evidence networks: a network meta-analysis in multiple myeloma
Source: BMC Med Res Methodol. 2018 Jun 28;18:66. doi: 10.1186/s12874-018-0509-7 (PMC6022299; doi:10.1186/s12874-018-0509-7)
Supplement: Supplementary file 6 — Rankogram. Shows the rankograms for the white and the black network of the RCT only analysis. (PDF 1103 kb) [file 12874_2018_509_MOESM6_ESM.pdf]

Rankogram for RCT only analysis

(a) White network

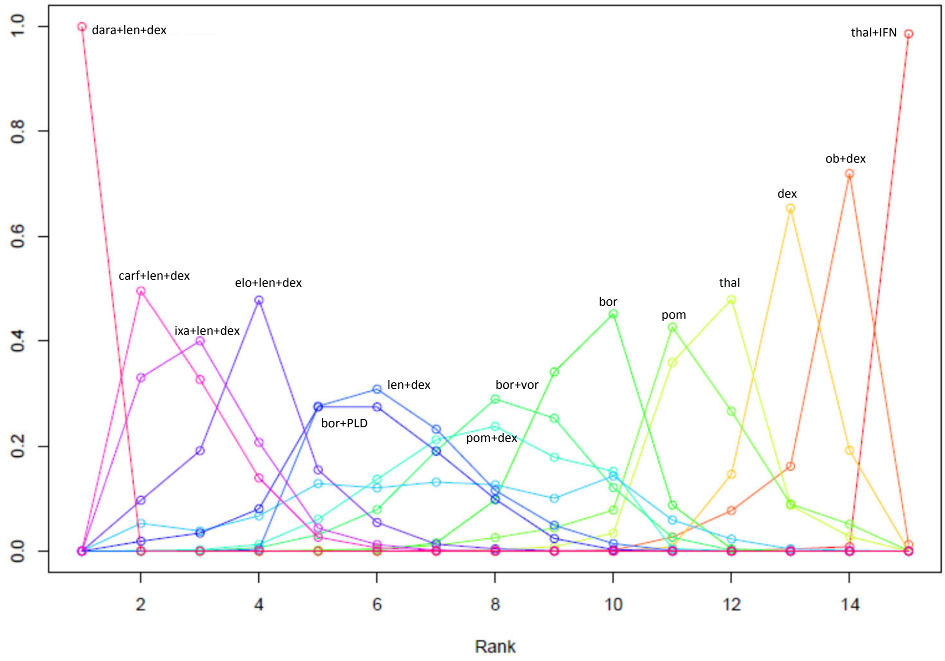

(b) Black network

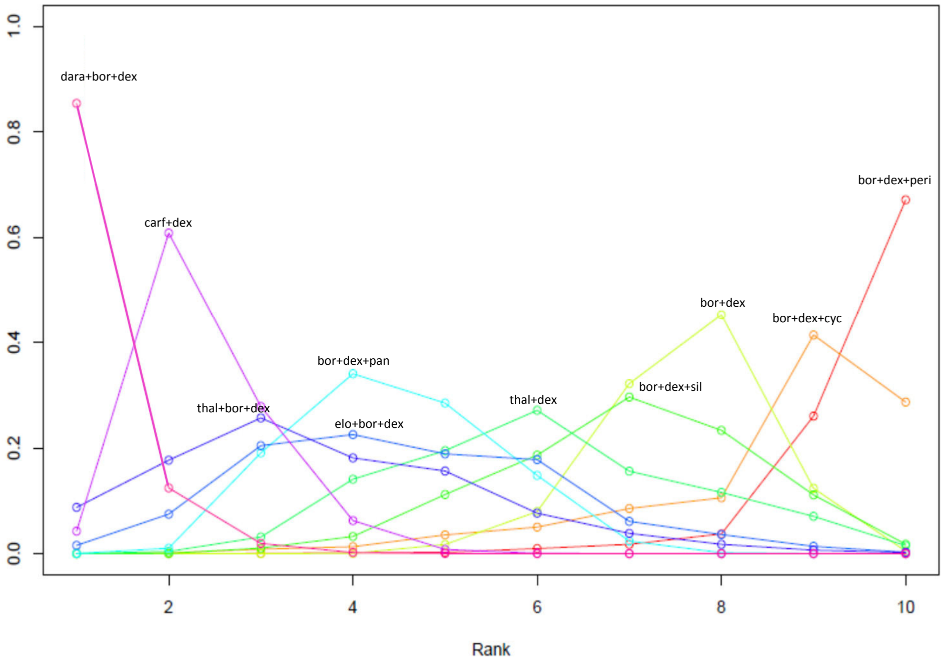

Additional File 7: all pairwise comparisons

	dara+bor+dex	carf+len+dex	ixa+len+dex	elo+len+dex	carf+dex	bor+PLD	len+dex	thal+bor+dex	elo+bor+dex	bor+bev	bor+dex+pan	pom+dex	bor+vor	thal+dex	bor	bor+dex+sil	bor+dex	pom	bor+dex+cyc	thal	bor+dex+peri	dex	ob+dex	thal+IFN
dara+len+dex	**0.56 (0.35,0.87)**	**0.51 (0.37,0.70)**	**0.49 (0.36, 0.67)**	**0.44 (0.32,0.62)**	**0.43 (0.28,0.65)**	**0.35 (0.22,0.53)**	**0.34 (0.27,0.43)**	**0.33 (0.18,0.62)**	**0.32 (0.17,0.58)**	**0.30 (0.16,0.59)**	**0.30 (0.20,0.44)**	**0.29 (0.19,0.42)**	**0.28 (0.18,0.44)**	**0.25 (0.15,0.42)**	**0.25 (0.17,0.37)**	**0.23 (0.14,0.38)**	**0.22 (0.15,0.32)**	**0.18 (0.10,0.32)**	**0.17 (0.08,0.35)**	**0.17 (0.11,0.26)**	**0.14 (0.07,0.26)**	**0.14 (0.10,0.19)**	**0.12 (0.07,0.20)**	**0.03 (0.01,0.11)**
dara+bor+dex		0.91 (0.58,1.42)	0.88 (0.56,1.36)	0.80 (0.50, 1.24)	0.77 (0.56,1.06)	0.62 (0.37,1.06)	**0.61 (0.40,0.90)**	0.60 (0.33,1.08)	**0.57 (0.34,0.96)**	0.55 (0.26,1.15)	**0.54 (0.39,0.74)**	**0.52 (0.32,0.83)**	**0.50 (0.29,0.84)**	**0.45 (0.28,0.73)**	**0.45 (0.28,0.72)**	**0.41 (0.27,0.63)**	**0.39 (0.30,0.50)**	**0.33 (0.18,0.60)**	**0.31 (0.16,0.58)**	**0.31 (0.18,0.53)**	**0.25 (0.14,0.44)**	**0.25 (0.16,0.39)**	**0.22 (0.12,0.39)**	**0.06 (0.02,0.20)**
carf+len+dex			0.97 (0.73,1.27)	0.87 (0.64,1.18)	0.85 (0.56,1.29)	0.68 (0.44,1.04)	**0.67 (0.55,0.82)**	0.66 (0.35,1.20)	0.62 (0.35,1.14)	0.60 (0.31,1.17)	**0.59 (0.41,0.85)**	**0.57 (0.39,0.83)**	**0.55 (0.36,0.84)**	**0.49 (0.30,0.82)**	**0.49 (0.34,0.71)**	**0.45 (0.27,0.74)**	**0.43 (0.29,0.61)**	**0.36 (0.21,0.61)**	**0.34 (0.17,0.68)**	**0.34 (0.23,0.51)**	**0.28 (0.15,0.52)**	**0.28 (0.21,0.37)**	**0.24 (0.15,0.39)**	**0.07 (0.02,0.22)**
ixa+len+dex				0.90 (0.67,1.22)	0.88 (0.58,1.30)	0.70 (0.47,1.07)	**0.69 (0.57,0.84)**	0.68 (0.37,1.24)	0.64 (0.36,1.15)	0.62 (0.32,1.22)	**0.61 (0.43,0.87)**	**0.59 (0.41,0.85)**	**0.57 (0.38,0.86)**	**0.51 (0.31,0.84)**	**0.51 (0.36,0.72)**	**0.47 (0.28,0.77)**	**0.44 (0.31,0.63)**	**0.37 (0.22,0.64)**	**0.35 (0.17,0.68)**	**0.35 (0.24,0.53)**	**0.28 (0.16,0.53)**	**0.28 (0.22,0.38)**	**0.25 (0.15,0.40)**	**0.07 (0.02,0.22)**
elo+len+dex					0.97 (0.63,1.50)	0.78 (0.51,1.22)	**0.77 (0.61,0.96)**	0.75 (0.41,1.39)	0.72 (0.40,1.31)	0.69 (0.35,1.36)	**0.68 (0.47,0.99)**	**0.65 (0.44,0.96)**	**0.63 (0.41,0.97)**	**0.57 (0.33,0.72)**	**0.56 (0.39,0.82)**	**0.52 (0.31,0.86)**	**0.49 (0.33,0.72)**	**0.41 (0.24,0.71)**	**0.39 (0.19,0.78)**	**0.39 (0.25,0.59)**	**0.32 (0.17,0.59)**	**0.32 (0.23,0.43)**	**0.27 (0.17,0.45)**	**0.07 (0.02,0.25)**
carf+dex						0.80 (0.49,1.32)	0.79 (0.55,1.13)	0.78 (0.44,1.37)	0.74 (0.45,1.21)	0.71 (0.35,1.46)	**0.70 (0.53,0.91)**	0.67 (0.43,1.06)	0.65 (0.40,1.06)	**0.58 (0.37,0.92)**	**0.58 (0.37,0.79)**	**0.54 (0.37,0.79)**	**0.50 (0.42,0.60)**	**0.42 (0.23,0.76)**	**0.40 (0.22,0.73)**	**0.40 (0.24,0.67)**	**0.32 (0.19,0.56)**	**0.33 (0.22,0.49)**	**0.28 (0.16,0.50)**	**0.08 (0.02,0.25)**
bor+PLD							0.98 (0.68,1.42)	0.97 (0.49,1.89)	0.91 (0.48,1.76)	0.88 (0.48,1.62)	0.87 (0.55,1.37)	0.83 (0.56,1.24)	0.81 (0.59,1.11)	0.72 (0.40,1.29)	**0.72 (0.58,0.91)**	0.67 (0.37,1.18)	**0.63 (0.39,0.99)**	**0.53 (0.30,0.92)**	0.49 (0.23,1.05)	**0.50 (0.33,0.76)**	**0.40 (0.20,0.81)**	**0.40 (0.30,0.55)**	**0.35 (0.21,0.58)**	**0.10 (0.03,0.31)**
len+dex								0.98 (0.55,1.74)	0.93 (0.55,1.74)	0.90 (0.48,1.69)	0.88 (0.66,1.20)	0.85 (0.62,1.16)	0.82 (0.57,1.20)	0.74 (0.46,1.18)	**0.74 (0.55,0.99)**	0.68 (0.43,1.06)	**0.64 (0.47,0.86)**	**0.54 (0.32,0.89)**	**0.50 (0.25,0.97)**	**0.51 (0.36,0.73)**	**0.41 (0.23,0.74)**	**0.41 (0.34,0.51)**	**0.36 (0.23,0.56)**	**0.10 (0.03,0.32)**
thal+bor+dex									0.95 (0.46,1.98)	0.91 (0.39,2.18)	0.90 (0.51,1.60)	0.86 (0.46,1.63)	0.84 (0.43,1.63)	0.75 (0.53,1.07)	0.75 (0.40,1.43)	0.69 (0.36,1.34)	0.65 (0.38,1.12)	0.55 (0.26,1.14)	0.51 (0.23,1.16)	0.52 (0.23,1.16)	**0.42 (0.20,0.89)**	**0.42 (0.23,0.76)**	**0.36 (0.18,0.76)**	**0.10 (0.03,0.36)**
elo+bor+dex										0.96 (0.42,2.25)	0.95 (0.57,2.25)	0.91 (0.49,1.69)	0.88 (0.46,1.71)	0.79 (0.41,1.47)	0.79 (0.43,1.46)	0.73 (0.41,1.30)	0.68 (0.43,1.08)	0.58 (0.28,1.19)	0.54 (0.26,1.15)	0.55 (0.28,1.19)	**0.44 (0.23,0.87)**	**0.44 (0.25,0.79)**	**0.38 (0.19,0.79)**	**0.10 (0.03,0.37)**
bor+bev											0.98 (0.49,1.96)	0.94 (0.49,1.83)	0.92 (0.50,1.70)	0.82 (0.38,1.79)	0.82 (0.46,1.44)	0.76 (0.35,1.67)	0.71 (0.35,1.43)	0.60 (0.28,1.28)	0.56 (0.22,1.38)	0.57 (0.29,1.10)	0.46 (0.19,1.12)	**0.46 (0.25,0.84)**	**0.40 (0.19,0.81)**	**0.11 (0.03,0.39)**
bor+dex+pan												0.96 (0.66,1.42)	0.93 (0.59,1.46)	0.84 (0.53,1.30)	0.83 (0.55,1.23)	0.77 (0.52,1.14)	**0.72 (0.60,0.88)**	0.61 (0.35,1.05)	0.57 (0.31,1.05)	**0.58 (0.37,0.92)**	**0.47 (0.27,0.80)**	**0.47 (0.33,0.66)**	**0.41 (0.24,0.68)**	**0.11 (0.03,0.36)**
pom+dex													0.97 (0.65,1.45)	0.87 (0.51,1.49)	0.87 (0.62,1.20)	0.80 (0.47,1.35)	0.75 (0.50,1.11)	**0.63 (0.43,0.94)**	0.60 (0.29,1.21)	**0.60 (0.41,0.90)**	**0.49 (0.25,0.93)**	**0.49 (0.37,0.63)**	**0.42 (0.26,0.68)**	**0.11 (0.03,0.38)**
bor+vor														0.90 (0.50,1.59)	0.89 (0.71,1.11)	0.82 (0.47,1.47)	0.77 (0.49,1.23)	0.65 (0.37,1.14)	0.61 (0.28,1.28)	**0.62 (0.40,0.94)**	**0.50 (0.25,0.99)**	**0.50 (0.37,0.69)**	**0.43 (0.26,0.71)**	**0.12 (0.04,0.39)**
thal+dex															1.00 (0.58,1.72)	0.92 (0.54,1.59)	0.86 (0.57,1.31)	0.73 (0.37,1.41)	0.68 (0.33,1.40)	0.69 (0.39,1.23)	0.56 (0.29,1.07)	**0.56 (0.34,0.92)**	**0.48 (0.26,0.91)**	**0.13 (0.04,0.46)**
bor																0.92 (0.55,1.56)	0.87 (0.58,1.30)	0.73 (0.44,1.23)	0.69 (0.33,1.41)	**0.69 (0.48,0.99)**	0.56 (0.29,1.09)	**0.56 (0.45,0.69)**	**0.49 (0.31,0.76)**	**0.13 (0.04,0.42)**
bor+dex+sil																	0.94 (0.67,1.31)	0.79 (0.42,1.52)	0.74 (0.38,1.48)	0.75 (0.43,1.34)	0.61 (0.33,1.13)	0.61 (0.37,1.00)	**0.53 (0.28,0.99)**	**0.14 (0.04,0.48)**
bor+dex																		0.84 (0.48,1.46)	0.79 (0.43,1.42)	0.80 (0.51,1.30)	0.65 (0.39,1.07)	**0.65 (0.46,0.93)**	**0.56 (0.33,0.96)**	**0.15 (0.05,0.50)**

pom	0.95 (0.55,1.66)	0.94 (0.55,1.66)	0.77 (0.36,1.64)	0.77 (0.48,1.21)	0.67 (0.36,1.24)	**0.18 (0.05,0.63)**
bor+dex+cyc		1.01 (0.48,2.15)	0.82 (0.37,1.77)	0.82 (0.41,1.64)	0.71 (0.32,1.58)	**0.19 (0.05,0.73)**
thal			0.81 (0.41,1.62)	0.81 (0.61,1.09)	0.70 (0.43,1.16)	**0.19 (0.06,0.58)**
bor+dex+peri				1.00 (0.55,1.87)	0.87 (0.42,1.81)	**0.24 (0.06,0.86)**
dex					0.87 (0.59,1.28)	**0.24 (0.07,0.75)**
ob+dex						**0.27 (0.08,0.91)**
