# Supplementary figures and images for: The use of single armed observational data to closing the gap in otherwise disconnected evidence networks: a network meta-analysis in multiple myeloma
Source: BMC Med Res Methodol. 2018 Jun 28;18:66. doi: 10.1186/s12874-018-0509-7 (PMC6022299; doi:10.1186/s12874-018-0509-7)

# SUCRA score all licensed treatments

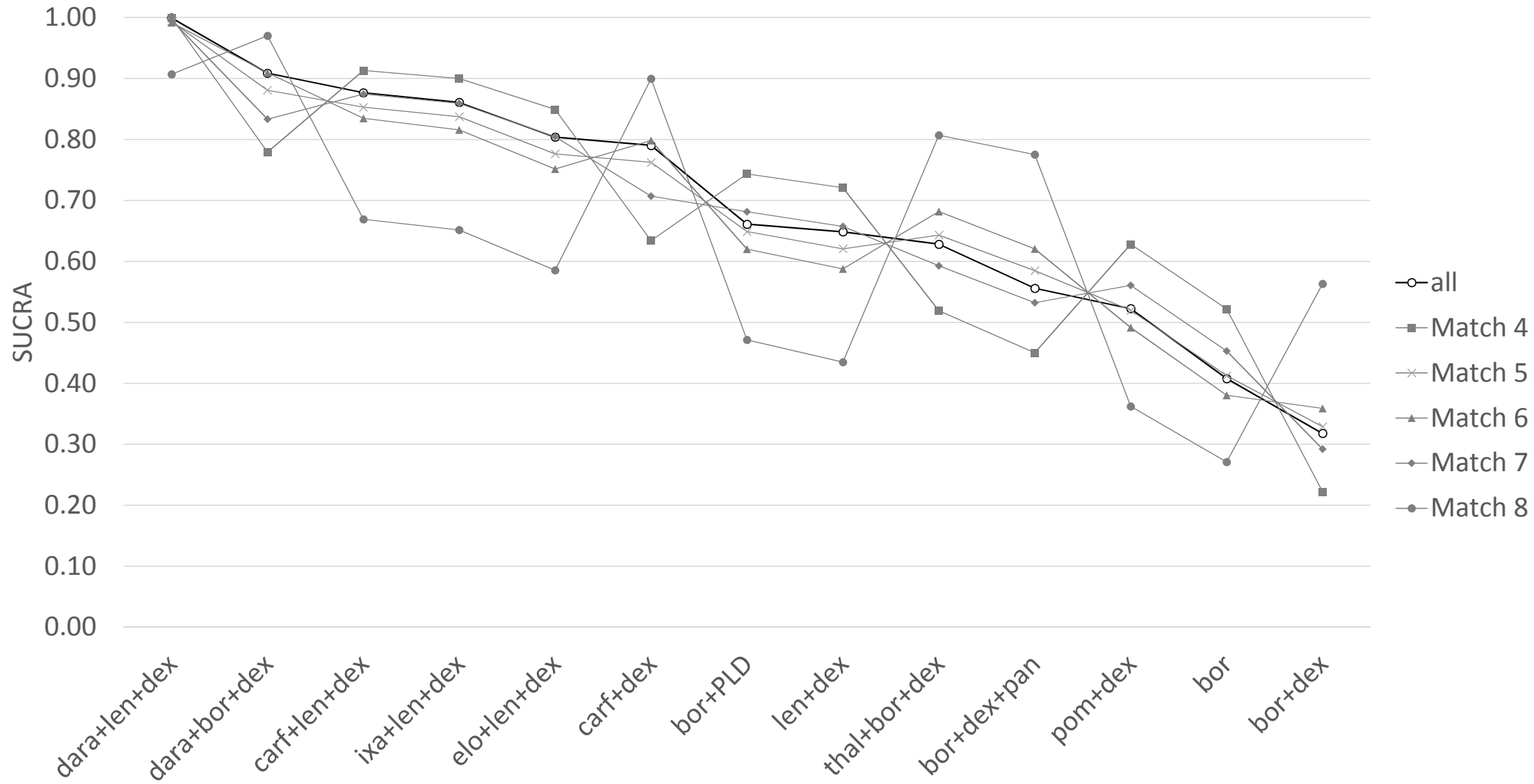

Supplement: Supplementary file 8 — SUCRA all scenarios. SUCRA ranking score of all licensed treatments of individual matches connecting both networks as well as the base case scenario containing all matches. (PDF 1100 kb) [file 12874_2018_509_MOESM8_ESM.pdf]
